# Diagnostic accuracy of an antigen-based point-of-care test versus nucleic acid amplification testing for genital trichomoniasis among pregnant women attending antenatal care facilities in Zambia

**DOI:** 10.1186/s12879-025-10698-9

**Published:** 2025-03-13

**Authors:** Sumire Sorano, Enesia Banda Chaponda, Massimo Mirandola, Ephraim Chikwanda, Vivian Mwewa, Joyce M. Mulenga, Mike Chaponda, Ludovica Ghilardi, Emma M. Harding-Esch, Chris Smith, Mitsuaki Matsui, Daniel Chandramohan, Daniel Schröder, Daniel Golparian, Mohamed Mahmoud Ali, Karel Blondeel, Magnus Unemo, Igor Toskin, R Matthew Chico

**Affiliations:** 1 Faculty of Infectious and Tropical Diseases, London School of Hygiene & Tropical Medicine London, London, WC1E 7HT United Kingdom; 2 School of Tropical Medicine and Global Health, Nagasaki University, 1-12-4 Sakamoto, Nagasaki, 852-8523 Japan; 3 Department of Biological Sciences, University of Zambia, Lusaka, Zambia; 4 Department of Diagnostics and Public Health, University of Verona, Verona, Italy; 5 Tropical Diseases Research Centre, Ndola, Zambia; 6 St. Paul’s Mission Hospital, Nchelenge, Zambia; 7 WHO Collaborating Centre for Sexually Transmitted Infections, London School of Hygiene & Tropical Medicine, London, UK; 8 Division of Global Health, Department of Public Health, Graduate School of Health Sciences Kobe University, Kobe, Japan; 9 Department of Laboratory Medicine, WHO Collaborating Centre for Gonorrhoea and Other STIs, Faculty of Medicine and Health, Örebro University, Örebro, Sweden; 10 Department of Sexual and Reproductive Health and Research, World Health Organization, Geneva, Switzerland; 11 Faculty of Medicine and Health Sciences, Ghent University, Ghent, Belgium; 12 Institute for Global Health, University College London (UCL), London, UK

**Keywords:** *Trichomonas vaginalis*, Point-of-care test, Pregnancy, Sexually transmitted infection (STI), Antenatal care (ANC)

## Abstract

**Background:**

Infection with *Trichomonas vaginalis* (TV) is the most prevalent curable sexually transmitted infection (STI) globally and is associated with prelabour rupture of membranes, preterm delivery, and low birthweight. Point-of-care (POC) testing for TV during pregnancy may facilitate rapid antenatal case detection and treatment. This study, part of the World Health Organization’s global ProSPeRo study, aimed to evaluate the performance of OSOM® Trichomonas Rapid Test, an antigen-based POC test, against a reference nucleic acid amplification test (NAAT) among pregnant women in Zambia. We also assessed the operational characteristics and patient acceptability of the POC test, within the context of WHO’s target product profiles for STI POC tests.

**Methods:**

We enrolled pregnant women attending four health centres in Nchelenge, Zambia, for antenatal care between 15 February and 26 May 2023. Vaginal swabs for the TV POC test and a reference NAAT (Aptima® *Trichomonas vaginalis* assay) were obtained. POC test results were read independently by two study staff members. Study staff filled a questionnaire on the operational characteristics of the POC test, and participants were asked about their willingness to wait for results.

**Results:**

Paired POC and reference test samples were collected from 1,015 participants. Overall, 23.0% (233/1015) tested positive for TV by NAAT, and 15.3% (155/1015) tested positive by the POC test, with three inconclusive results. The overall sensitivity and specificity of the POC test were 66.4% (95% confidence intervals [CI] 57.7–74.1%) and 99.6% (95% CI: 98.8–99.9%), respectively. Sensitivity was higher among those with TV-associated symptoms compared to those without (83.6% versus 60.4%, relative ratio 1.39, 95% CI 1.14–1.68). Inter-rater agreement was 99.7% (Cohen’s Kappa 0.989). The study staff (*n* = 14) found the test easy to use and interpret, with most staff (12/14) reporting results were available within 25 min.

**Conclusion:**

Overall, the TV POC test showed lower sensitivity than WHO’s 85% target, but exceeded the 99% specificity target. Among symptomatic pregnant women, sensitivity nearly reached the WHO target. The assay was user-friendly, required minimal training, and delivered results quickly. Further studies are needed to determine the optimal antenatal settings for this technology.

**Trial registration:**

PACTR202302766902029.

**Supplementary Information:**

The online version contains supplementary material available at 10.1186/s12879-025-10698-9.

## Introduction

Infection with the parasite *Trichomonas vaginalis* (TV) is the most prevalent curable sexually transmitted infection (STI) in the world [1]. A systematic review and meta-analysis showed that TV infection during pregnancy was associated with pre-labour rupture of membranes, preterm delivery, and low birthweight [2]. In sub-Saharan Africa, the pooled prevalence estimate of TV among pregnant women has been reported to be 13.8% [3]. Wet mount microscopy or culture have been frequently used to diagnose trichomoniasis but both require a microscope and other sophisticated laboratory equipment, and have poor sensitivity and specificity when compared to nucleic acid amplification tests (NAATs) [4]. Syndromic algorithms have been developed to aid in the management of non-viral STIs in low-resource settings. However, because trichomoniasis is often asymptomatic in women, syndrome-based management of STIs has limited utility. In antenatal care (ANC) settings, the sensitivity of these algorithms for diagnosing TV with vaginal discharge is low, whereas specificity is higher [5–8]. Point-of-care (POC) tests for TV are available and may facilitate rapid antenatal case detection and subsequent treatment [4]. This is particularly important given time-sensitive nature of pregnancy [9]. Although there is uncertainty regarding the value of screening all pregnant women [10], a targeted approach could benefit women with specific risk factors or conditions, requiring further investigation. POC tests should, ideally, meet the REASSURED criteria (**R**eal-time connectivity, **E**ase of specimen collection, **A**ffordable, **S**ensitive, **S**pecific, **U**ser-friendly, **R**apid and robust, **E**quipment free or simple and environmentally friendly, **D**eliverable to end-users) [11].

The World Health Organization (WHO) recently published target product profiles for POC tests for syphilis, gonorrhoea, chlamydial infection and trichomoniasis [12]. In this context, we evaluated the performance of an antigen-based test for TV (OSOM® Trichomonas Rapid Test, Sekisui Diagnostics, LLC, Massachusetts, USA) when compared to a reference NAAT (Aptima® *Trichomonas vaginalis* assay; Hologic, San Diego, USA) for detection of TV in pregnant women in Nchelenge, Zambia. Aptima® *Trichomonas vaginalis* assay uses transcription-mediated amplification targeting ribosomal RNA, and is highly sensitive and specific [13]. Additionally, we assessed the operational characteristics of the POC test among study staff and its acceptability to the study participants. The OSOM® Trichomonas Rapid Test kits have a Clinical Laboratory Improvement Amendments waiver from the United States Food and Drug Administration and have been used in STI clinics and as part of sexual and reproductive services. The test uses an immunochromatographic capillary flow based “dipstick technology” that incorporates a pair of murine monoclonal antibodies. One antibody is conjugated to particles and dried onto the dipstick, whereas the other is immobilised on the dipstick’s surface. If present, TV antigens will bind to the primary anti-trichomonas antibody conjugated to coloured particles, forming a complex. The complex is then captured by a second anti-trichomonas antibody coated on the nitrocellulose membrane, to produce a visible blue test line. To the best of our knowledge, this antigen-based POC test has not been evaluated as a general screening tool for pregnant women in the antenatal setting. Our study is part of the WHO global ProSPeRo study (Project on STI POC Testing). This specific study was also registered with the Pan African Clinical Trials Registry (PACTR202302766902029, dated 13/09/2022) and this report was prepared in accordance with the Standards for Reporting Diagnostic Accuracy (STARD) guidelines [14].

## Methods

### Sample size calculations

The sample size was calculated with the method described in the global ProSPeRo standard protocol [15] using an expected TV prevalence of 24.8% based on a pregnancy cohort study conducted between 2013 and 2014 in the same facilities of the Nchelenge District [16]. Assumptions regarding the sensitivity and specificity of the OSOM® Trichomonas Rapid Test were derived from published studies [17–24] (Supplementary Table 1), the majority of which had tested symptomatic women. The sensitivity and specificity were estimated to be 80% and 98%, respectively. Thus, to produce prevalence estimates within an accuracy of 5% based on a 2-sided alpha level of 0.05, we determined that 253 positive cases were needed to estimate sensitivity and 26 negative cases were required for specificity. Under the assumption of 24.8% prevalence, with an additional 10% to accommodate potential withdrawals or censoring from analysis, the total sample size required was 1,021.

### Study site

This study was implemented in four health centres that provide ANC services in the Nchelenge District of Zambia: Kabuta, Kafutuma, Kashikishi, and Nchelenge. All health centres are within the catchment area of the Saint Paul’s Mission Hospital in Luapula Province, northern Zambia, along the eastern shore of Lake Mweru with a total population of approximately 233,000 inhabitants [25].

### Study participants

Pregnant women at a minimum of 13 gestational weeks who attended the designated clinics for ANC services were recruited into the study. Individuals were excluded if they had received treatment with metronidazole or clindamycin during their current pregnancy, had a known allergy or if treatment with metronidazole was contraindicated, or had used a vaginal cream or ointment product, douched, or used vaginal lubricants within 72 h of recruitment.

### Procedures

**Fig. 1:**
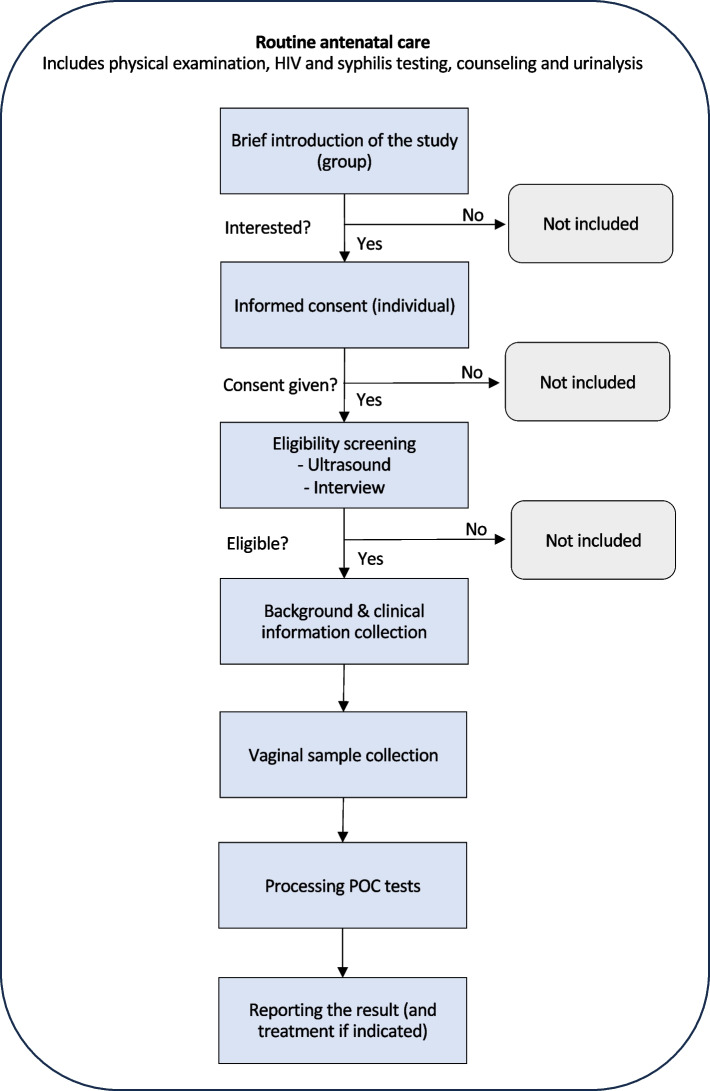
Participant flow diagram of antigen-based point-of-care test for trichomoniasis among pregnant women in Zambia

The participant flow in the study is illustrated in Fig. 1. After obtaining written informed consent from prospective participants, gestational age was determined by sonography as part of the eligibility screening. For pregnant adolescents under 18 years of age, we obtained assent from the patients themselves, followed by consent from their parents in compliance with Zambian law. For eligible participants, background and clinical information including self-reported TV-associated symptoms and other STI symptoms (unusual vaginal discharge, pain during urination, itching or burning of the vulva) was collected. We asked questions regarding patients’ willingness to wait for POC test results. We recorded this information in an electronic case report form designed in REDCap [26].

Clinical staff collected four vaginal swabs per participant: one for the TV POC test, one for the bacterial vaginosis (BV) POC test that was being evaluated in parallel [data to be presented elsewhere], one for NAAT analysis of TV, and one for Nugent scoring for BV. The order of swab collection was randomised so equal numbers of women provided swabs throughout the study in the 24 variations of order that are possible when collecting four samples per participant. Taking this into consideration, we generated a random list of 1,100 sampling sequences prior to study initiation. An independent researcher, otherwise not involved in the study, prepared opaque envelopes containing the sample collection order and participant IDs. When eligibility was determined, participants opened the envelopes when their eligibility was determined, to reveal the order of swabbing to be followed. POC tests were performed according to manufacturers’ instructions by clinical or laboratory staff. Results were then independently read by two clinicians, or one clinician and one laboratory staff member, each of whom entered the results into REDCap, with neither staff member knowing the reading of the other. A separate study staff member then communicated the POC test results to participants in writing. If participants tested positive for either TV or BV, study staff administered a 2-g dose of metronidazole at the same visit. At the end of the study, a questionnaire was completed by the clinic staff about the operational utility characteristics of the POC test.

### Laboratory analysis

The Aptima® *Trichomonas vaginalis* Assay was used as the reference standard NAAT. Rayon swabs in the kit were used for sample collection. We placed the samples in a cooler box filled with ice and transferred to Saint Paul’s Mission Hospital for storage at −80℃ within six hours of collection. Samples were later shipped to the WHO Collaborating Centre for Gonorrhoea and Other STIs at Örebro University in Sweden for reference testing. Clinical information and results of the POC tests were blinded to those who performed the reference NAAT in Sweden. HIV and syphilis tests were conducted as part of routine antenatal care. Core tests® ONE STEP Syphilis Test Kit (Core Technology, Atlanta, USA) was used for the screening of syphilis, and Determine® HIV Test Kit (Abbott, Illinois, USA) was used for HIV screening.

### Data handling

Data were directly entered into REDCap by the study staff using electronic tablets with consistency and validation rules to prevent data entry error. The study coordinator reviewed daily entries, uploading records through a protected portal to a secure server hosted by the London School of Hygiene & Tropical Medicine in the United Kingdom. The research team reviewed records weekly and resolved data queries. Frequency tables were generated to assess completeness and accuracy of data collected throughout enrolment.

### Statistical analysis

Basic characteristics of participants were described using numbers and proportions for categorical variables, and the median and interquartile range (IQR) for continuous variables. POC test results (positive, negative, indeterminate, or invalid) were compared with results from the Aptima® *Trichomonas vaginalis* Assay as a reference standard. Any indeterminate or invalid results, as well as instances of missing data, were excluded from the analysis of accuracy measures; we reported the number of invalid results separately. Two-by-two tables were constructed for each facility, based on the results of the POC test for TV and the NAAT reference test, so that the four cells represented true positives, false negatives, false positives, and true negatives. Sensitivity, specificity, positive and negative likelihood ratios (LR), positive and negative predictive values (PPV and NPV) were estimated by comparing the index test with the reference laboratory test using a bivariate random effect meta-analysis model with logit transformation [27] to address both within-site variation and between-site variations. Stata midas and metaDTA software packages [28–30] were used for the meta-analysis. This modelling took into account potential threshold effects and the correlation between sensitivity and specificity. Sub-group analyses were conducted based on presence of TV-associated symptoms to see if diagnostic accuracy estimates differed depending on the presence or absence of symptoms as pre-specified in the protocol. Based on prevalence scenarios, positive and negative LRs were plotted using Fagan’s nomogram in order to obtain post-test infection probability. Observed inter-rater agreement and Cohen’s Kappa were calculated. The numbers and proportion of each category of answers obtained from the operational characteristics and the willingness to wait questions were calculated. We used Stata 18.0 software [31] for our analyses.

## Results

**Fig. 2:**
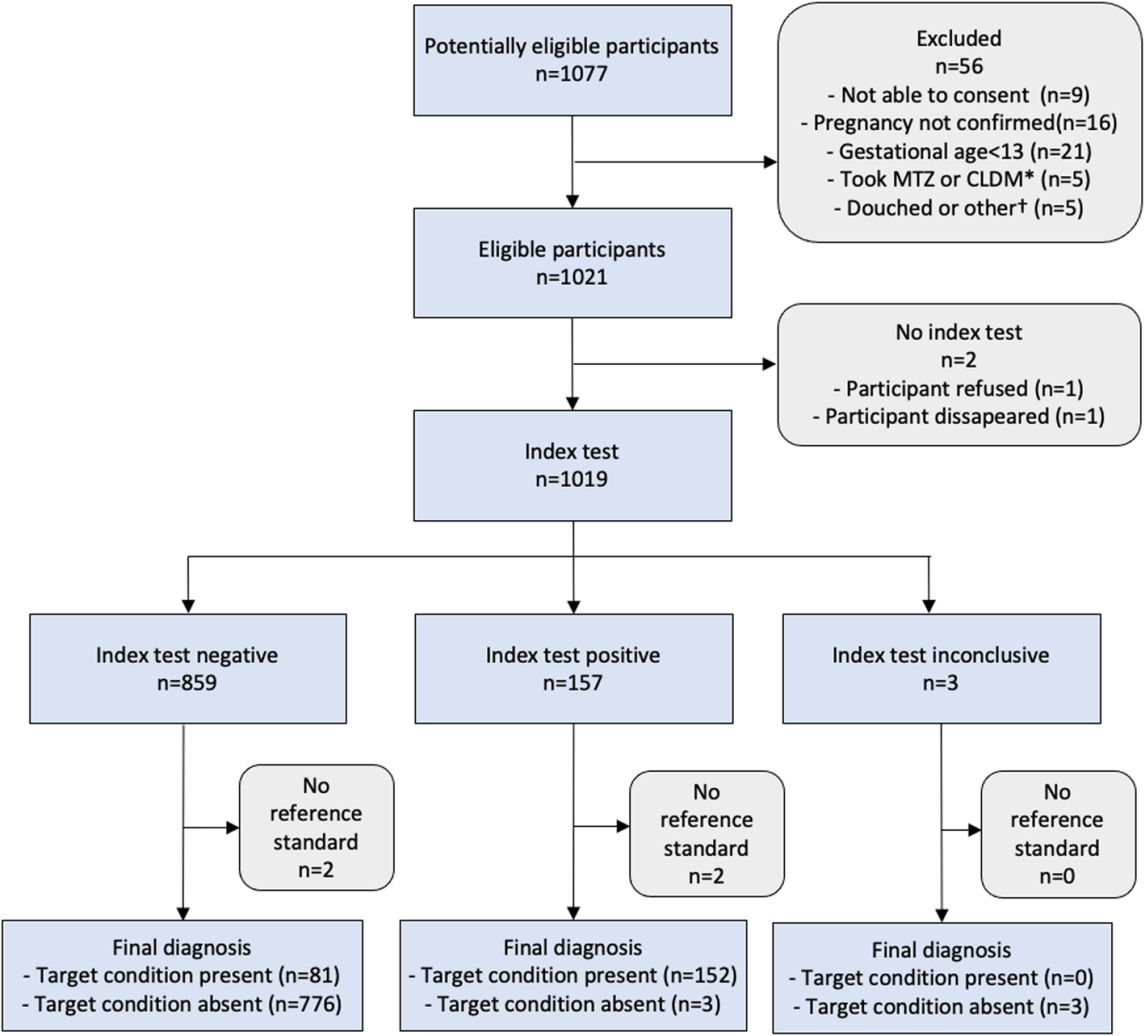
STARD 2015 flow diagram. * Used metronidazole (MTZ) or clindamycin (CLM) during current pregnancy; † Used or performed any of the following within 72 h of visit: (i) vaginal cream or ointment product, (ii) douching, or (iii) spermicides, vaginal lubricants, or feminine sprays

Between February 15 and May 26, 2023, 1,077 potentially eligible individuals were screened for study entry, and 1,021 of these were enrolled in the study. Results of both the TV POC test and the reference NAAT test were obtained in 1,015 individuals. The STARD 2015 flow diagram is shown in Fig. 2. The median participant age was 23 years (IQR: 20–30) (Table 1). The majority, (70.7%), of pregnant women were married or living with a partner, whereas remaining participants (29.3%) were single, separated, divorced, or widowed. In total, 56.9% of women were in their second trimester (13–27 gestational weeks), whereas 43.1% were in their third trimester (28–39 gestational weeks). Nineteen percent had at least one TV-associated symptom. The most common symptom was itching or burning of the vulva (15.3%) and 6.2% reported having an unusual vaginal discharge. A total of 8.6% tested HIV-positive and 14.7% were seropositive for syphilis using a treponemal POC test.

**Table 1:** Basic characteristics of participating pregnant women in Zambia, 2023

Basic characteristics (*N* = 1,015)	Number or median	Percentage or IQR
**Facility**		
Kabuta	138	13.6%
Kafutuma	144	14.2%
Kashikishi	451	44.4%
Nchelenge	282	27.8%
**Age (years)**	23	20–30
**Age (years) in category**		
< 18	36	3.6%
18–24	551	54.3%
25–34	323	31.8%
35 and above	105	10.3%
**Marital status**		
Single	288	28.4%
Married or living with a partner	717	70.7%
Separated, divorced, or widowed	9	0.9%
**Number of previous pregnancies**		
0	310	30.5%
1	225	22.2%
2	176	17.3%
3 or more	304	30.0%
**Gestational week at enrolment**		
13–27 week	578	56.9%
28–39 week	437	43.1%
**Symptoms associated with trichomoniasis**		
Unusual vaginal discharge^a^	63	6.2%
Pain during urination^a^	43	4.2%
Itching or burning of the vulva^a^	155	15.3%
At least one symptom above^b^	196	19.3%
**HIV status**		
Positive	87	8.6%
Negative	918	90.4%
Unknown	10	1.0%
**Syphilis point-of-care test**		
Positive	149	14.7%
Negative	862	84.9%
Unknown	4	0.4%

^a^One participant was missing value

^b^Two participants were missing value

**Fig. 3:**
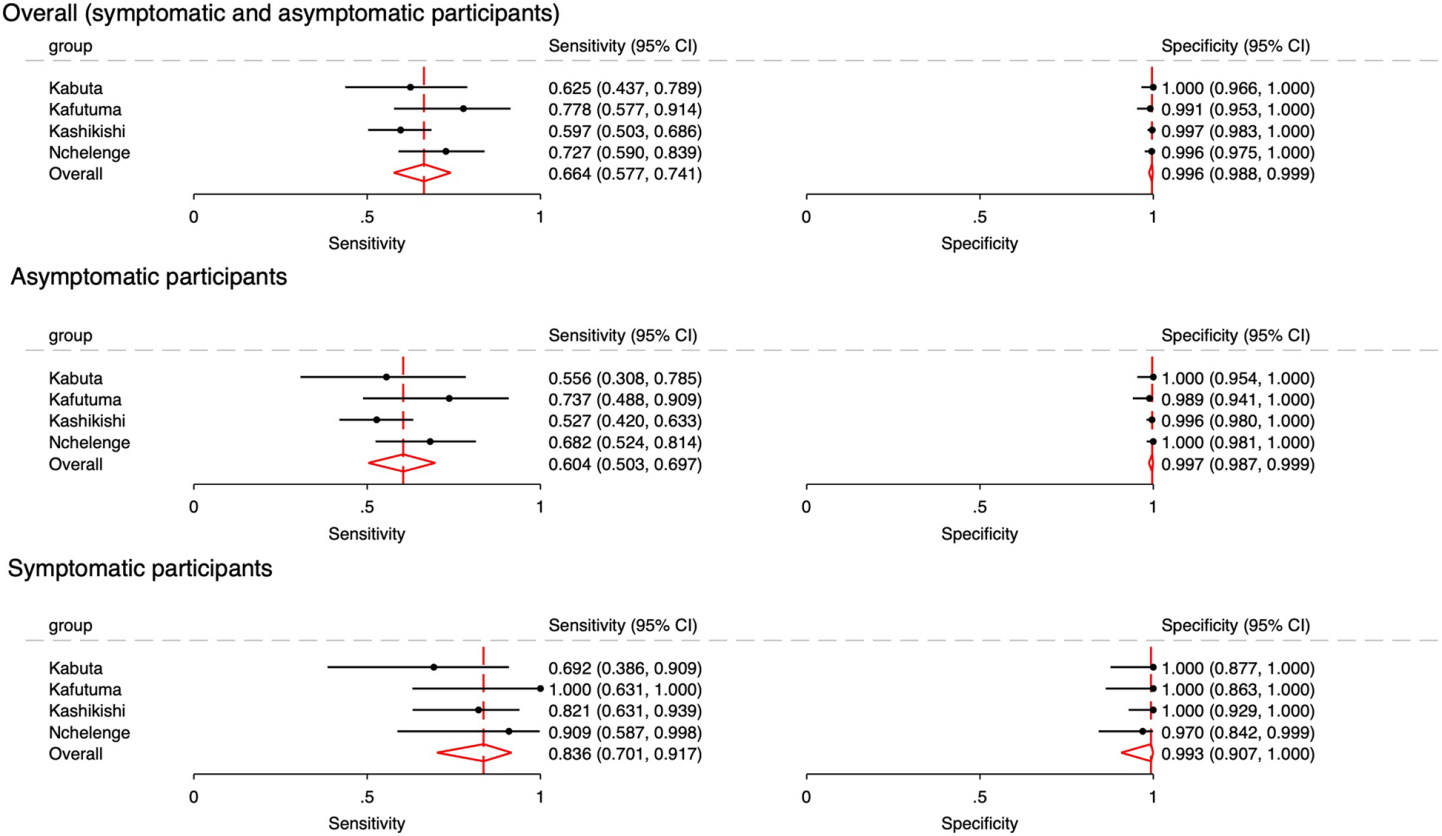
Forest plots of sensitivity and specificity of OSOM® Trichomonas Rapid Test compared to reference NAAT (Aptima® *Trichomonas vaginalis* Assay) for *T. vaginalis* in pregnant women in Zambia, 2023

The results of the POC test and NAAT for TV at each facility are presented in Table 2, whereas the prevalence estimates based on the NAAT and accuracy measures of the antigen-based POC test for TV are shown in Table 3. Prevalence estimates by NAAT were 23.2%, 18.8%, 26.4%, and 19.6% in Kabuta, Kafutuma, Kashikishi, and Nchelenge, respectively. Three samples produced indeterminate or invalid POC test results, all of which were negative by NAAT. Figure 3 shows the forest plots of sensitivity and specificity of the POC test. The specificities of the POC were uniformly high across facilities, ranging between 99.1% and 100.0%. In contrast, sensitivities ranged from 59.7% in Kashikishi to 77.8% in Kafutuma. The pooled prevalence, sensitivity and specificity across facilities were 22.3% (95% CI 18.4–26.2%), 66.4% (95% CI 57.7–74.1%), 99.6% (95% CI 98.8–99.9%), respectively.

**Table 2:** Results of OSOM® Trichomonas Rapid Test and reference NAAT (Aptima® *Trichomonas vaginalis* Assay) for *T. vaginalis* in pregnant women in Zambia, 2023 (*n* = 1,015)

Site	Index test result	NAAT for *Trichomonas vaginalis*		
		**Condition present**	**Condition absent**	**Total**
**Kabuta**				
Positive		20	0	20
Negative		12	106	118
Indeterminate or invalid		0	0	0
Total		32	106	138
**Kafutuma**				
Positive		21	1	22
Negative		6	116	122
Indeterminate or invalid		0	0	0
Total		27	117	144
**Kashikishi**				
Positive		71	1	72
Negative		48	330	378
Indeterminate or invalid		0	1	1
Total		119	332	451
**Nchelenge**				
Positive		40	1	41
Negative		15	224	239
Indeterminate or invalid		0	2	2
Total		55	227	282
**Combined**				
Positive		152	3	155
Negative		81	776	857
Indeterminate or invalid		0	3	3
Total		233	782	1015

**Table 3:** Participant characteristics and performance of OSOM® Trichomonas Rapid Test compared to reference NAAT (Aptima® *Trichomonas vaginalis* Assay) for *T. vaginalis* in pregnant women in Zambia, 2023

Participant characteristics	*N*	Prevalence (%) (95% CI)	Sensitivity (%) (95% CI)	Specificity (%) (95% CI)
**Overall (symptomatic and asymptomatic participants)**				
Kabuta	138^a^	23.2 (16.4 – 31.1)	62.5 (43.7 – 78.9)	100.0 (96.6 –100.0)
Kafutuma	144	18.8 (12.7 – 26.1)	77.8 (57.7 – 91.4)	99.1 (95.3 – 100.0)
Kashikishi	450^a^	26.4 (22.4 – 30.8)	59.7 (50.3 – 68.6)	99.7 (98.3 – 100.0)
Nchelenge	280	19.6 (15.2 – 24.8)	72.7 (59.0 – 83.9)	99.6 (97.5 – 100.0)
Pooled estimate	1,012^a^	22.3 (18.4 – 26.2)	66.4 (57.7 – 74.1)	99.6 (98.8 – 99.9)
Positive likelihood ratio = 171.2 (54.2 – 541.1)				
Negative likelihood ratio = 0.34 (0.26 – 0.43)				
Diagnostic odds ratio = 508 (156 – 1655)				
**Asymptomatic participants**				
Kabuta	96^a^	18.8 (11.5 – 28.0)	55.6 (30.8 – 78.5)	100.0 (95.4 – 100.0)
Kafutuma	111	17.1 (10.6 – 25.4)	73.7 (48.8 – 90.9)	98.9 (94.1 – 100.0)
Kashikishi	371^a^	24.5 (20.2 – 29.2)	52.7 (42.0 – 63.3)	99.6 (98.0 – 100.0)
Nchelenge	236	18.6 (13.9 – 24.2)	68.2 (52.4 – 81.4)	100.0 (98.1 – 100.0)
Pooled estimate	814^a^	20.4 (16.7 – 24.0)	60.4 (50.3 – 69.7)	99.7 (98.7—99.9)
Positive likelihood ratio = 195.2 (46.8 – 813.8)				
Negative likelihood ratio = 0.40 (0.31 – 0.51)				
Diagnostic odds ratio = 491 (112 – 2159)				
**Symptomatic participants**				
Kabuta	41^a^	31.7 (18.1 – 48.1)	69.2 (38.6 – 90.9)	100.0 (87.7 – 100.0)
Kafutuma	33	24.2 (11.1 – 42.3)	100.0 (63.1 – 100.0)	100.0 (86.3 – 100.0)
Kashikishi	78^a^	35.9 (25.3 – 47.6)	82.1 (63.1 – 93.9)	100.0 (92.9 – 100.0)
Nchelenge	44	25.0 (13.2 – 40.3)	90.9 (58.7 – 99.8)	97.0 (84.2 – 99.9)
Symptomatic	196^a^	30.1 (23.7 – 36.5)	83.6 (70.1 – 91.7)	99.3 (90.7—100.0)
Positive likelihood ratio = 124.0 (8.2 – 1881.8)				
Negative likelihood ratio = 0.17 (0.09 – 0.32)				
Diagnostic odds ratio = 749 (39 – 14,320)				

Symptomatic participants had at least one of the following: unusual vaginal discharge, pain during urination, itching or burning of the vulva

^a^The sum of symptomatic and asymptomatic cases is not equal to the overall count; one case each in Kabuta and Kashikishi was missing symptom information

The TV positivity detected by TV NAAT was higher among individuals with TV-associated symptoms compared to those without these symptoms (30.1% [95%CI 23.7 – 36.5] vs 20.4% [95%CI 16.7 – 24.0]; relative ratio 1.48, 95% CI 1.14 – 1.89; *P* = 0.0026). The POC sensitivity was also significantly higher among individuals with these symptoms compared to those without (83.6% [95%CI 70.1 – 91.7] versus 60.4% [95%CI 50.3 – 69.7], relative ratio 1.39, 95% CI 1.14–1.68; *P* = 0.0001). The PPVs and NPVs based on prevalence scenarios of the minimum, maximum of the observed site and pooled prevalence are shown in Table 4. Overall, PPVs ranged from 97.6% to 98.4%, and NPVs from 89.4% to 92.7%. Fagan’s nomogram is plotted based on prevalence scenario and positive and negative LR (Additional file 1 Figures S1-S3). Observed inter-rater agreement of POC test results was 99.7% (1,016/1,019), with a Cohen’s Kappa of 0.989.

**Table 4:** Positive predictive value and negative predictive value based on prevalence scenarios

Prevalence range (%)	Pooled sensitivity (%)	Pooled specificity (%)	Prevalence scenarios	PPV (95%CI)	NPV (95%CI)
**Overall (symptomatic and asymptomatic participants)**					
22 (19 – 26)	66.4	99.6	19	97.6 (92.7 – 99.2)	92.7 (90.8 – 94.2)
			22	98.0 (93.9 – 99.3)	91.3 (89.1 – 93.1)
			26	98.4 (95.0 – 99.5)	89.4 (86.8 – 91.5)
**Asymptomatic participants**					
20 (17—25)	60.4	99.7	17	97.6 (90.6 – 99.4)	92.5 (90.6 – 94.0)
			20	98.0 (92.1 – 99.5)	91.0 (88.7 – 92.8)
			25	98.5 (94.0 – 99.6)	88.3 (85.5 – 90.6)
**Symptomatic participants**					
30 (24—36)	83.6	99.3	24	97.5 (72.1 – 99.8)	95.0 (90.9 – 97.3)
			30	98.2 (77.8 – 99.9)	93.4 (88.1 – 96.4)
			36	98.6 (82.1 – 99.9)	91.5 (84.9 – 95.4)

Prevalence scenarios are based on the minimum and maximum of the observed site and pooled prevalence

*PPV* Positive predictive value, *NPV* Negative predictive value, *95%CI* 95% Confidence interval

The acceptability component involved asking two questions: (i) *If these rapid tests are available at this clinic in the future, would you be willing to wait for the results at the clinic, directly after the tests are performed?* And (ii) *If yes, how long would you be willing to wait?* Nearly all women, 98.7% (1,000/1,013), said they would be willing to wait for POC test results at the facility if the same devices were available in the future. Just 1.2% (12/1,013) responded saying they would not wait, and one woman said she did not know if she would wait. Of those who said they would be willing to wait, 3.2% (32/1,000) were willing to wait for up to 2 h, 24.4% (245/1,000) were willing to wait for up to 1 h, 60,2% (602/1,000) up to 30 min, 99.4% (994/1,000) up to 20 min, and 0.6% (6/1,000) of respondents did not know. All participants of the study did wait for the POC test results.

The responses of the study staff recorded following administration of their questionnaire regarding the operational/utility characteristics of the OSOM® Trichomonas Rapid Test are shown in Table 5. Overall, the antigen-based POC test showed high levels of acceptability among both clinical and laboratory staff.

**Table 5:** Assessment of operational characteristics of the OSOM® Trichomonas Rapid Test by study staff (*n* = 14)

Operational characteristics	Clinicians	Laboratory staff	Total	Percentage
Number of staff interviewed	8	6	14	100.0%
Clarity of kit instructions				
Difficult to follow	0	0	0	0.0%
Fairly clear	0	0	0	0.0%
Very clear	6	4	10	71.4%
Excellent	2	2	4	28.6%
Ease of use				
Complicated	0	0	0	0.0%
Fairly easy	0	0	0	0.0%
Very easy	7	5	12	85.7%
Excellent	1	1	2	14.3%
Ease of interpretating results				
Difficult	0	0	0	0.0%
Fairly easy	0	1	1	7.1%
Very easy	3	4	7	50.0%
Unambiguous	5	1	6	42.9%
Rapidity of test results				
< 15 min	3	0	3	21.4%
15–25 min	3	6	9	64.3%
> 25 min	2	0	2	14.3%
Hands-on time				
< 5 min	3	0	3	21.4%
5–10 min	2	4	6	42.9%
> 10 min	3	2	5	35.7%
Training time required				
< 30 min	4	2	6	42.9%
30 min—1 h	4	4	8	57.1%
> 1 h	0	0	0	0.0%
Number of tests needed to be performed before being comfortable with POC test				
1	2	2	4	28.6%
2	3	0	3	21.4%
3	2	2	4	28.6%
4	0	0	0	0.0%
5	0	2	2	14.3%
6 or more	1	0	1	7.1%

## Discussion

In these ANC settings in Zambia, the antigen-based POC test for TV demonstrated moderate sensitivity at 66.4%, and high specificity at 99.6% when used to screen a population that included both symptomatic and asymptomatic individuals during pregnancy. Overall, the POC test did not meet the WHO target for sensitivity of 85% among POC tests for TV. However, the POC test exceeded the WHO target for specificity (99%) [11]. Among symptomatic participants, the sensitivity increased to 83.6%, almost reaching the WHO’s target. The overall sensitivity of the POC test in this study was at the lower end of the range compared to past studies which used a NAAT as reference standard (67—92%), while specificity was within the reported range (97–100%) [17–24] (Additional file 1 Table S1). Past studies targeted populations predominantly with STI symptoms, and pregnant women had not been included. This was the first study to evaluate the POC test among pregnant women in an ANC setting which included both symptomatic and asymptomatic individuals. Therefore, it is likely that the performance characteristics of the TV POC test could vary depending on the ratio of symptomatic to asymptomatic individuals in the ANC population being screened. In our study, 19.3% had TV-associated symptoms.

The WHO target product profile for TV POC tests state that training time should require less than 90 min, and, when deployed, test results should be available within 60 min and easily interpretable with minimal instructions [12]. The OSOM® Trichomonas Rapid Test met all these criteria. Moreover, the WHO target product profile emphasizes the importance of POC tests that can be used by health care providers without dependency on laboratory technicians. In our study, swabs were collected by clinicians, and the POC test results were interpreted either by two clinicians, or by one clinician and one laboratory staff member, and we used the results of clinicians for the diagnostic accuracy analysis. Importantly, study staff who read the POC test results were blinded to each other’s reading. In this context, we found a very high inter-rater agreement of 99.7%.

We shipped all samples from Zambia to Sweden at the end of the study, and the longest any sample had been stored before analysis by NAAT was six months. The storage conditions were optimal throughout: −80 °C from the day of collection in Zambia and between—60 °C and −80 °C in transit to Sweden before processing. Thus, storage time is unlikely to have compromised the reference NAAT testing for any samples.

The reference test, Aptima *Trichomonas vaginalis* Assay, utilises transcription-mediated amplification technology, which targets ribosomal RNA. There are thousands of ribosomal RNA copies within a single cell, allowing the assay to detect even minimal protozoa loads. This could explain why the sensitivity of the TV POC test was found to be low in our study, although our findings in the sub-group of symptomatic cases were consistent with prior studies of symptomatic women. Further investigation is needed, such as quantifying TV in the samples with quantitative PCR (qPCR) or performing viability PCR and comparing these results to the POC test results.

There are other potential sources of bias that merit consideration. The prevalence of TV in this study may have been influenced by the antenatal booking procedures of each facility. Participants were recruited on a fixed day of the week at each facility, and within each facility there was a designated day for the first ANC visit. Consequently, the gestational age of pregnant women at some facilities was more advanced than at others. Additionally, the random order of collecting four vaginal swabs from each participant was aimed at balancing the overall biological material collected. While this approach could have affected sensitivity, specificity, PPV and NPV estimates, any potential impact on results would likely have been minimal, particularly given the increase in vaginal discharge women experience during pregnancy.

From an operational standpoint, the TV POC test was user-friendly, requiring minimal training and delivered results within 25 min in the majority of cases. Given the rural African context of this study, where pregnant women travel long distances to attend ANC health centres, the POC test, although lacking in sensitivity, resulted in the treatment of many women who would otherwise have remained undiagnosed. The high specificity of the test, and thus the high PPV, is indicative of relatively little overtreatment when the POC test would be used in the field.

In our study, samples were collected by clinical staff. However, some studies have demonstrated that self-collected swabs can achieve comparable sensitivity and specificity to clinician-collected samples. Self-collection offers several advantages, including increased privacy, accessibility, and convenience, making it an attractive option. To validate fully the use of POC tests with self-collected swabs and maximize their potential impact, further research is needed to assess their performance under such conditions.

## Conclusion

As a tool for general screening of pregnant women for TV, this antigen-based POC test did not meet the WHO target for sensitivity, but it exceeded the WHO target for specificity. The POC test performed better in the sub-group of pregnant women who were symptomatic, nearly reaching the WHO target for sensitivity, and exceeding the WHO target for specificity. Further investigation is needed to understand which antenatal settings and sub-groups might benefit from this technology.

## Supplementary Information

Additional file 1: Diagnostic accuracy of antigen-based point-of-care versus nucleic acid amplification testing for genital trichomoniasis among pregnant women attending antenatal care facilities in Zambia

## Data Availability

The datasets used and/or analysed during the current study are available from the corresponding author on reasonable request.

## Abbreviations

ANC: Antenatal care
CI: Confidence interval
IQR: Interquartile range
LR: Likelihood ratio
NAAT: Nucleic acid amplification test
NPV: Negative predictive value
POC: Point-of-care
PPV: Positive predictive value
ProSPeRo study: Project on Sexually Transmitted Infection Point-of-care Testing
RR: Risk ratio
STI: Sexually transmitted infection
TV: *Trichomonas vaginalis*
WHO: World Health Organization
