# Supplementary material for: Diagnostic accuracy of an antigen-based point-of-care test versus nucleic acid amplification testing for genital trichomoniasis among pregnant women attending antenatal care facilities in Zambia
Source: BMC Infect Dis. 2025 Mar 13;24(Suppl 1):1482. doi: 10.1186/s12879-025-10698-9 (PMC11905427; doi:10.1186/s12879-025-10698-9)
Supplement: Supplementary file 1 — Additional file 1: Diagnostic accuracy of antigen-based point-of-care versus nucleic acid amplification testing for genital trichomoniasis among pregnant women attending antenatal care facilities in Zambia [file 12879_2025_10698_MOESM1_ESM.docx]

**Additional file 1**

**Diagnostic accuracy of antigen-based point-of-care versus nucleic acid amplification testing for genital trichomoniasis among pregnant women attending antenatal care facilities in Zambia****;** Sumire Sorano, Enesia Banda Chaponda, Massimo Mirandola, Ephraim Chikwanda, Vivian Mwewa, Joyce M Mulenga, Mike Chaponda, Ludovica Ghilardi, Emma M Harding-Esch, Chris Smith, Matsui Mitsuaki, Daniel Chandramohan, Daniel Schröder, Daniel Golparian, Mohamed Mahmoud Ali, Karel Blondeel, Magnus Unemo, Igor Toskin, R Matthew Chico

**Table S1.** Published studies using OSOM® Trichomonas Rapid Test among women which use NAAT for reference standard

| **Author and publication year** | **Country** | **Year of study** | **Sample size** | **Symptomatic** | **Pregnant** | **Prevalence (%)** | **Reference test** | **Sensitivity (%)** | **Specificity (%)** | **Samples collected by whom** | **Test performed by whom** |
| --- | --- | --- | --- | --- | --- | --- | --- | --- | --- | --- | --- |
| Piwonka 2016 [15] | United States | NR | 99 | Yes | No | 25.3 | Xpert TV PCR | 84.0 | 100 | NR | NR |
| Huppert 2010 [16] | United States | 2006-2008 | 209 | Yes: 74%  No: 26% | No | 24.4 | CRS: Culture + Aptima | 86.3 | 100 | Clinician | Clinician |
| Territo 2016 [17] | United States | 2011-2012 | 188 | Yes | No | 17.6 | NAAT (no detail available) | 69.7 | 100 | Clinician | Clinician or research staff |
| Verwijs 2019 [18] | Rwanda | 2016-2017 | 690 | Yes: 86%  No: 14% | No | 16.1 | In-house PCR | 68.5 | 97.4 | Clinician | NR |
| Pillay 2004  [19] | United States | NR | 428 | Yes | No | 12.6 | CRS: Wet mount +  in-house PCR | 66.7 | 100 | Clinician | Laboratory staff |
| Jones 2013 [20] | South Africa | NR | 230 | Yes: 27%  No: 73% | No | 10.4 | In-house PCR | 83.3 | 96.6 | Self | Self |
| Jones 2013 [20] | Brazil | NR | 695 | Yes: 75%  No: 25% | No | 2.7 | In-house PCR | 68.4 | 99.9 | Self | Self |
| Nathan 2014 [21] | United Kingdom | 2011-2012 | 246 | Yes | No | 9.8 | ≧2 positive with WP, culture, OSOM, In-house PCR, Aptima | 91.7 | 100 | Clinician | Trained technician |
| Garret 2018 [22] | South Africa | 2016-2017 | 247 | Yes: 88.4%  No: 11.6% | No | 3.2 | Anyplex II STI-7 | 75 | 100 | Clinician | NR |

NR: not reported; CRS: composite reference standard; WP: wet preparation examination = wet mount; NAAT: Nucleic acid amplification test

**
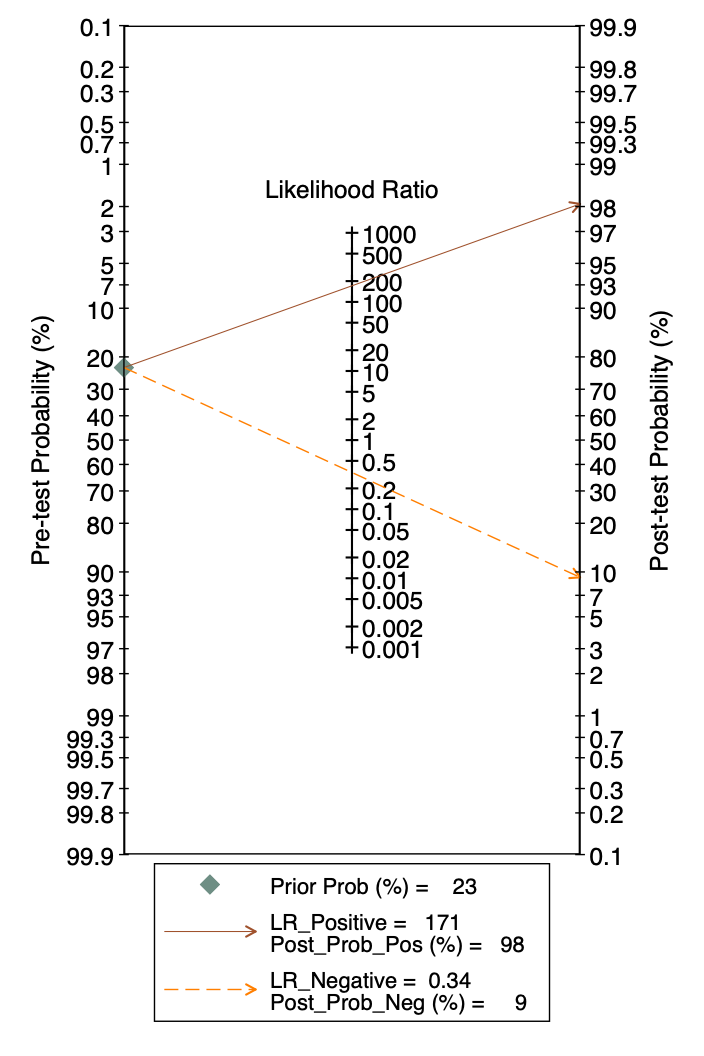
**

**Figure S1.** Fagan’s nomogram demonstrating diagnostic utility of *Trichomonas vaginalis* POC test among pregnant women (both symptomatic and asymptomatic) attending antenatal care in Zambia

**
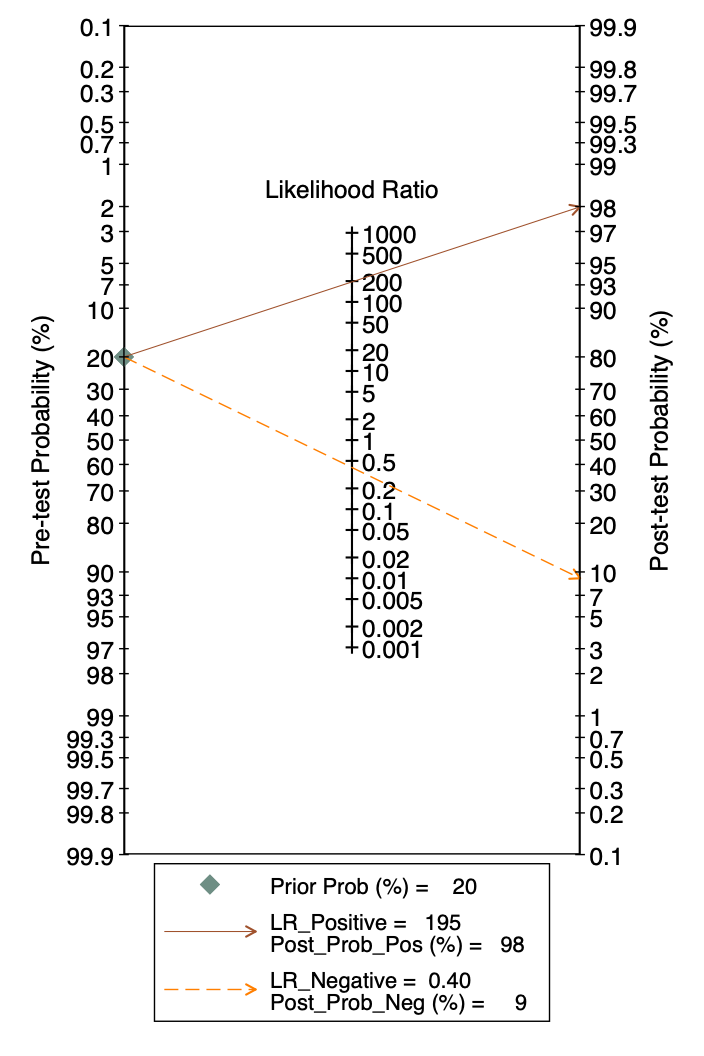
**

**Figure S2.** Fagan’s nomogram demonstrating diagnostic utility of *Trichomonas vaginalis* POC test among asymptomatic pregnant women attending antenatal care in Zambia

**
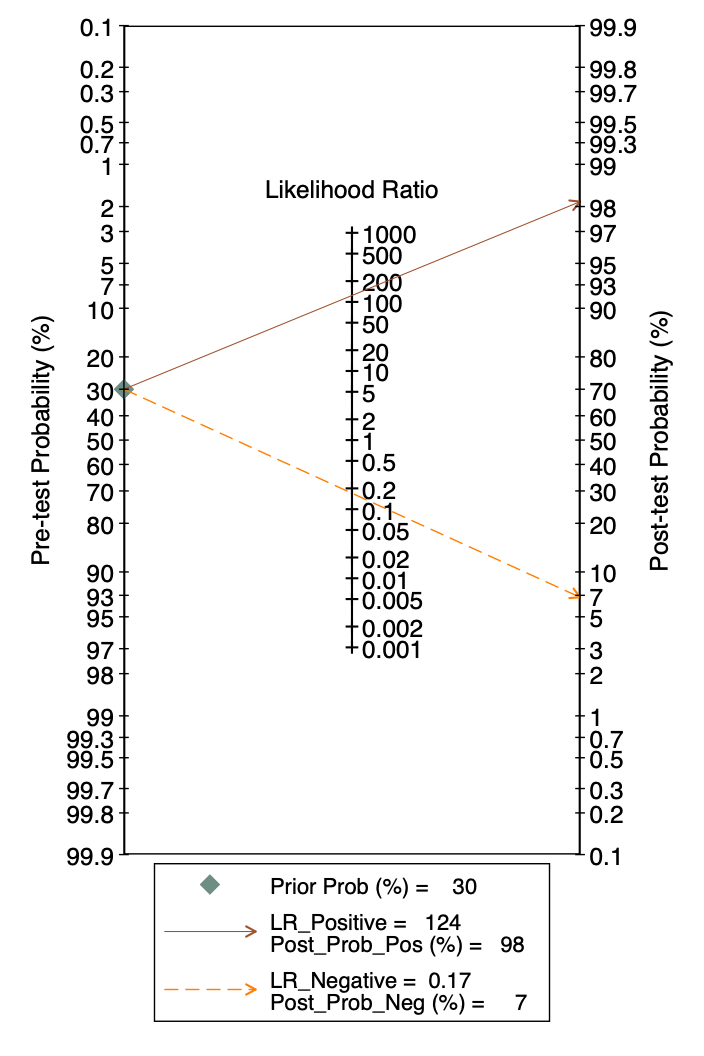
**

**Figure S3.** Fagan’s nomogram demonstrating diagnostic utility of *Trichomonas vaginalis* POC test among symptomatic pregnant women attending antenatal care in Zambia
